# Lumbar puncture-verified subarachnoid hemorrhage: bleeding sources, need of radiological examination, and functional recovery

**DOI:** 10.1007/s00701-023-05640-4

**Published:** 2023-05-25

**Authors:** Rozerin Kevci, Anders Lewén, Elisabeth Ronne-Engström, Fartein Velle, Per Enblad, Teodor Svedung Wettervik

**Affiliations:** Department of Medical Sciences, Section of Neurosurgery, Uppsala University, SE-751 85 Uppsala, Sweden

**Keywords:** Clinical outcome, Intracranial aneurysm, Lumbar puncture, Neurointensive care, Subarachnoid hemorrhage

## Abstract

**Background:**

The primary aim was to determine the diagnostic yield of vascular work-up, the clinical course during neurointensive care (NIC), and rate of functional recovery for patients with computed tomography (CT)-negative, lumbar puncture (LP)-verified SAH.

**Methods:**

In this retrospective study, 1280 patients with spontaneous SAH, treated at our NIC unit, Uppsala University Hospital, Sweden, between 2008 and 2018, were included. Demography, admission status, radiological examinations (CT angiography (CTA) and digital subtraction angiography (DSA)), treatments, and functional outcome (GOS-E) at 12 months were evaluated.

**Results:**

Eighty (6%) out of 1280 SAH patients were computed tomography (CT)-negative, LP-verified cases. Time between ictus and diagnosis was longer for the LP-verified SAH cohort in comparison to the CT-positive patients (median 3 vs 0 days, *p* < 0.001). One fifth of the LP-verified SAH patients exhibited an underlying vascular pathology (aneurysm/AVM), which was significantly less common than for the CT-verified SAH cohort (19% vs. 76%, *p* < 0.001). The CTA- and DSA-findings were consistent in all of the LP-verified cases. The LP-verified SAH patients exhibited a lower rate of delayed ischemic neurological deficits, but no difference in rebleeding rate, compared to the CT-verified cohort. At 1-year post-ictus, 89% of the LP-verified SAH patients had recovered favorably, but 45% of the cases did not reach good recovery. Having an underlying vascular pathology and an external ventricular drainage were associated with worse functional recovery (*p* = 0.02) in this cohort.

**Conclusions:**

LP-verified SAH constituted a small proportion of the entire SAH population. Having an underlying vascular pathology was less frequent in this cohort, but still occurred in one out of five patients. Despite the small initial bleeding in the LP-verified cohort, many of these patients did not reach good recovery at 1 year, this calls for more attentive follow-up and rehabilitation in this cohort.

**Supplementary Information:**

The online version contains supplementary material available at 10.1007/s00701-023-05640-4.

## Introduction

Subarachnoid hemorrhage (SAH) represents 5% of all stroke cases and carries a high burden of mortality and morbidity [11, 18, 45]. If SAH due to aneurysmal rupture (aSAH) is left untreated, clinical outcome is often poor due to complications including rebleeding, acute hydrocephalus, and delayed ischemic neurological deficits (DIND). One key aspect in patient management is early diagnosis and treatment in order to mitigate the risks of these secondary complications [14, 30, 37]. The diagnostic work-up for SAH includes at first an emergency non-contrast head computed tomography (CT) and, if negative, a lumbar puncture (LP) more than 6-12 hours after ictus, to analyze the presence of blood-break-down products [4, 9]. In the last years, there has been a debate about the necessity to proceed with LP, since CT has a sensitivity and specificity near 100%, if performed within six hours after ictus and assessed by an experienced radiologist [2, 6, 23, 41]. However, although rupture of an intracranial aneurysm usually leads to an extensive amount of SAH that is visible on CT, small bleeds also occur. Particularly, if the time from ictus to CT is long, the SAH could have already been flushed away and diluted in the cerebrospinal fluid (CSF) making it not visible on CT. In addition, experienced radiologists may not be available at all hospitals [6, 10, 34]. Therefore, the Swedish guidelines recommend to proceed with LP in case of high suspicion of SAH despite a negative CT within 6 hours from ictus [47]. Another consideration, after LP-verified SAH has been diagnosed, is the methods of choice for detection of any underlying vascular pathology (aneurysm, arteriovenous malformation (AVM)) by further radiological imaging, i.e., CT angiography (CTA) and/or digital subtraction angiography (DSA) [26]. Since a LP-verified SAH means a relatively small bleeding, the risk of having an underlying vascular pathology may be lower. However, the literature has reported a wide range of positive findings within 5–49% in LP-verified SAH [3, 7, 8, 13, 19, 42, 48]. Thus, it remains challenging to decide on the extent of vascular work-up.

Previous studies have mainly focused on determining the diagnostic yield of LP and the role of early CT alone to confirm SAH. In the present study, we aimed to investigate the clinical characteristics, the rate and risk factors for detecting an underlying vascular pathology, as well as the long-term functional outcome for patients with LP-verified SAH.

## Materials and methods

### Patients and study design

This retrospective study was conducted at the Department of Neurosurgery, Uppsala University Hospital, Sweden. The Department provides neurosurgical care for a central part of Sweden, with a catchment population around 2 million people. There were 1325 patients with spontaneous SAH, treated between January 7^th^ 2008 and April 9^th^ 2018, who were eligible for inclusion in this study. For the 1325 patients, 45 were excluded; 33 patients were treated at another neurointensive care (NIC) unit for more than 3 days during the first 10 days, 11 patients had missing clinical and radiological data, and 1 patient was younger than 16 years of age. The final study cohort was hence 1280 SAH patients, and 80 of those had a CT-negative LP-verified SAH (Supplementary fig. 1).

### Clinical management

Patients with either a CT- or LP-positive SAH within our catchment area were admitted to our NIC or neurointermediate care unit. LP-verified SAH was defined as pathologically elevated blood-break-down products (oxyhemoglobin, bilirubin) and not just visual inspection (xanthochromia) of or high erythrocyte levels in the CSF. Most of these cases were admitted from a local hospital within our catchment area. To localize the source of bleeding, a CTA was performed first, both in CT- and LP-verified cases. CTA was usually considered sufficient in peri-mesencephalic SAH cases if the first CT had been done early after ictus (within 24 h). CTA was also usually considered sufficient if SAH was verified with LP early after ictus. When the CTA was performed > 24 h after ictus in peri-mesencephalic SAH cases and LP-verified cases, a DSA was in general conducted in addition to the negative CTA. When the CTA was negative in cases with more extensive SAH, a DSA was also performed. In case of positive CTA findings for an underlying vascular pathology, this was considered sufficient work-up for some aneurysm cases, but usually a complimentary DSA was also done to better characterize the pathology before decision of treatment method. In cases with a high suspicion of aneurysmal rupture, but negative CTA and DSA, one or more examinations with CTA and/or DSA were performed within a few days to a week in between for re-evaluation.

The general NIC management of patients with SAH has been described in detail in previous studies [33, 38]. Unconscious patients were intubated and mechanically ventilated. Aneurysms were treated early by endovascular intervention or clipping to avoid rebleeding. All patients with CT-verified SAH were prescribed nimodipine for 3 weeks, but this was terminated in perimesencephalic SAH if the vascular imaging did not diagnose an aneurysm. DIND was defined as a clinical neurological deterioration in consciousness or development of focal neurological deficits, after other causes such as hydrocephalus, rebleeding, and meningitis had been excluded. In intubated patients, neurological wake-up tests were done six times per day for timely detection of any neurological deterioration. In case of DIND, patients were given triple-H therapy [15]. External ventricular drains (EVD) were used for intracranial pressure monitoring and CSF drainage. Thiopental coma and/or decompressive craniectomy (DC) were used as last-tier treatments in case of refractory intracranial hypertension.

### Data collection and analysis

Demographic, admission status, and treatment variables during NIC were collected. The World Federation of Neurosurgical Societies (WFNS) scale was used to determine the neurological condition at admission [46]. The Fisher scale was used to classify and evaluate the amount of SAH on the initial head CT [29]. Time to diagnosis was defined as the time in days between ictus and diagnosis of SAH.

### Functional outcome

Specially trained personnel evaluated the functional outcome at 12 months post-ictus by a structured telephone interview with the patient (if they had recovered) or their next-of-kin, using the Extended Glasgow Outcome Scale (GOS-E). GOS-E contains eight categories of outcome—from death (1) to upper good recovery (8) [20]. GOS-E was dichotomized as favorable/unfavorable outcome (GOS-E 5-8/1-4).

### Statistical analysis

Demography, admission variables, treatments, and clinical outcome were described as numbers (proportion) for categorical data and as medians (interquartile range (IQR)) for continuous data. Differences in these variables were analyzed between the CT- and LP-verified SAH groups, using the Mann-Whitney *U*-test or the Pearson’s Chi-square test, depending on the type of data.

A univariate logistic regression was done to determine the odds ratio (OR) and 95% confidence interval (CI) for having a DSA-verified vascular pathology, with age, sex, WFNS grade, and time from ictus to diagnosis, respectively, as the explanatory variables, in the LP-verified SAH cohort. The Spearman rank correlation test was used to evaluate the association between GOS-E and potentially explanatory variables, including age, sex, WFNS grade, time from ictus to diagnosis, vascular pathology, length of stay in the NIC/neurointermediate care, and rebleeding in the LP-verified SAH group.

A *p*-value < 0.05 was considered statistically significant. All statistical analyses were conducted in software program SPSS Statistics version 27 (IBM Corp., Armonk, NY, USA).

## Results

### Demography, admission variables, and treatments

Out of 1280 patients with spontaneous SAH, 80 (6%) of these were CT-negative, LP-verified cases (Table 1) and the remaining 1200 were CT-positive cases. Those with a LP-verified SAH were younger (median (IQR) 53 years (41–64) vs. 59 years (50–68), *p* < 0.001) and exhibited a lower WFNS grade (median (IQR) 1 (1-1) vs. 2 (1–4), *p* < 0.001). The LP-verified SAH patients were diagnosed at a later stage from ictus than those with CT-verified SAH (median (IQR) 3 (1–6) vs. 0 (0–1) days, *p* < 0.001). Among the 80 LP-verified SAH patients, 15 (19%) had a vascular pathology (Tables 1 and 2). Thirteen of these were intracranial aneurysms (ACom=9, ICA=3, and the basilar tip=1) and two of these were arteriovenous malformations (AVM). These vascular pathologies were seen both on CTA and DSA (Tables 1 and 2). For the remaining 65 patients with a negative CTA, 48 had a negative DSA and in 17 cases a DSA was not performed (Table 2). Among these 17 patients, 8 patients had a time between ictus and diagnosis of less than 24 hours.

**Table 1:** Demography, admission variables, treatments, and clinical outcome in relation SAH-diagnosis (CT- or LP-verified)

	All patients	CT-verified SAH	LP-verified SAH	*p*
Patients, *n* (%)	1280 (100%)	1200 (94%)	80 (6%)	N/A
Age, median (IQR) years	59 (50-68)	59 (50-68)	53 (41-64)	***<0.001***
Sex (female/male), *n* (%)	810/470 (63/37%)	760/440 (63/37%)	50/30 (63/38%)	0.88
Pupillary abnormality, n (%)	33 (3%)	33 (3%)	0 (0%)	0.13
WFNS grade, median (IQR)	1 (1-4)	2 (1-4)	1 (1-1)	***<0.001***
Fisher grade, median (IQR)	3 (2-4)	3 (3-4)	1 (1-1)	***<0.001***
Time to diagnosis, median (IQR) days	1 (0-1)	0 (0-1)	3 (1-6)	***<0.001***
Bleeding source detected with CTA (aneurysm/AVM/none/not performed), *n* (%)	874/12/372/22 (68/1/29/2%)	861/10/307/22 (72/1/26/2%)	13/2/65/0 (16/3/81/0%)	***<0.001***
Bleeding source detected with DSA (aneurysm/AVM/none/not performed),* n* (%)	735/15/284/246 (57/1/22/19%)	722/13/236/229 (60/1/20/19%)	13/2/48/17 (16/3/60/21%)	***<0.001***
Vascular treatment for patients with a vascular pathology (endovascular/surgical/both/none), *n* (%)	660/188/10/63 (72/20/1/7%)	645/188/10/63 (71/21/1/7%)	15/0/0/0 (100/0/0/0%)	0.11
Rebleeding, *n* (%)	80 (6%)	78 (7%)	2 (3%)	0.15
Delayed ischemic neurologic deficit, *n* (%)	212 (17%)	212 (18%)	0 (0%)	***<0.001***
Thiopental, *n* (%)	67 (5%)	66 (6%)	1 (1%)	0.10
Decompressive craniectomy, *n* (%)	57 (5%)	57 (5%)	0 (0%)	***0.05***
External ventricular drainage,* n* (%)	610 (48%)	607 (51%)	3 (4%)	***<0.001***
Favorable/unfavorable outcome, *n* (%)	727/553 (57/43%)	656/544 (55/45%)	71/9 (89/11%)	***<0.001***

*GCS M* Glasgow Come Scale Motor Score, *WFNS* Word Federation of Neurosurgical Societies, *IQR* interquartile range, *CTA* computed tomography angiography, *DSA* digital subtraction angiography, *AVM* arteriovenous malformation, *N/A* not applicable

*p*-values in bold and italics indicate statistical significance

**Table 2:** CTA- and DSA-findings

	DSA-findings				
CTA-findings	Negative	Aneurysm	AVM	Not performed	Total
LP-verified SAH patients					
Negative	48 (74%)	0 (0%)	0 (0%)	17 (26%)	65 (100%)
Aneurysm	0 (0%)	13 (100%)	0 (0%)	0 (0%)	13 (100%)
AVM	0 (0%)	0 (0%)	2 (100%)	0 (0%)	2 (100%)
Not performed	0 (0%)	0 (0%)	0 (0%)	0 (0%)	0 (100%)
CT-verified SAH patients					
Negative	233 (76%)	20 (7%)	3 (1%)	51 (16%)	307 (100%)
Aneurysm	3 (0.3%)	702 (82%)	0 (0%)	156 (18%)	861 (100%)
AVM	0 (0%)	0 (0%)	10 (100%)	0 (0%)	10 (100%)
Not performed	0 (0%)	0 (0%)	0 (0%)	22 (100%)	22 (100%)

*AVM* arteriovenous malformation. *CT* computed tomography. *CTA* CT angiography. *DSA* digital subtraction angiography. *LP* lumbar puncture. *SAH* subarachnoid hemorrhage

The LP-verified patients with a diagnosed vascular pathology were treated with occlusion of the vascular pathology in all cases (100%). Among the CT-verified SAH patients with a diagnosed vascular pathology, embolization/clipping was done in 93% of the cases. Thiopental (1% vs. 6%, *p* = 0.10) and DC (0% vs. 5%, *p* = 0.05) were less frequently used in the LP-verified SAH group as compared to the CT-verified group. Rebleeding occurred at a slightly lower rate (3% vs. 7%, *p* = 0.15) compared with the CT-verified SAH cases. In the LP-verified SAH cohort, the two rebleedings that occurred were in patients with a vascular pathology (both with an ACom aneurysm). There were no reported cases of late rebleeding after the acute phase among the patients with LP-verified SAH. There was no case of DIND in the LP-verified SAH group (0% vs. 18%), *p* < 0.001). EVD was used in a substantially higher frequency among CT-verified patients (51% vs. 4%, *p* < 0.001).

### Risk factors for a vascular pathology on DSA after LP-verified SAH

**Fig. 1:**
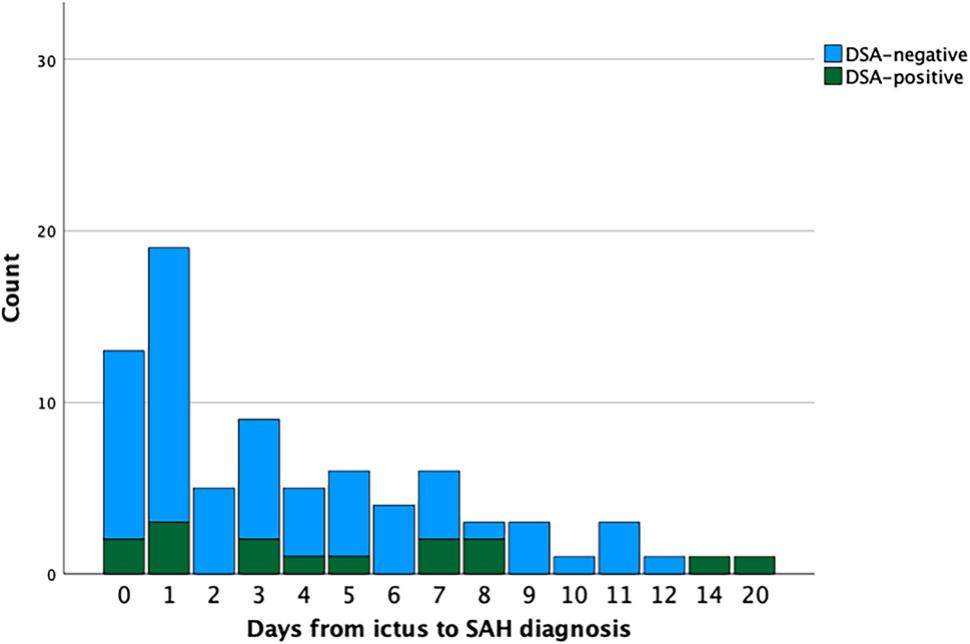
Time from ictus to SAH diagnosis in relation to DSA-findings among LP-verified SAH patients. The stacked bar chart represents the number of days from ictus to SAH diagnosis in the LP-verified SAH cohort in relation to DSA-findings. Although the majority of cases with an early diagnosis (before day 4) were DSA-negative, there were still a few DSA-positive cases during this time. DSA = digital subtraction angiography. LP = lumbar puncture. SAH = subarachnoid hemorrhage

In the LP-verified SAH cohort, age, sex, neurological status at admission (WFNS), and time from ictus to SAH diagnosis were not associated with the risk to find a vascular pathology on DSA, in univariate logistic regression analyses (Table 3). There was a trend towards a higher rate of positive DSA-findings when the time between ictus and diagnosis was longer (OR (95% CI) = 1.13 (0.98 -1.29), *p* = 0.09), but some of the LP-verified patients who were diagnosed within the first 48 h were also found to have an underlying vascular pathology (Fig. 1).

**Table 3:** Risk factors for a pathological DSA-finding after LP-verified SAH – a univariate logistic regression analysis of unfavorable outcome

Variable	Odds ratio (95%CI)	*p*
Age (years)	0.99 (0.95-1.03)	0.64
Sex (female)	1.83 (0.53-6.38)	0.34
WFNS (grade)	2.01 (0.68-5.94)	0.21
Time to diagnosis (days)	1.13 (0.98 -1.29)	0.09

*CI* confidence interval. *DSA* digital subtraction angiography. *LP* lumbar puncture. *SAH* subarachnoid hemorrhage. *WFNS* World Federation of Neurosurgical Societies

### LP-verified SAH and functional outcome

**Fig. 2:**
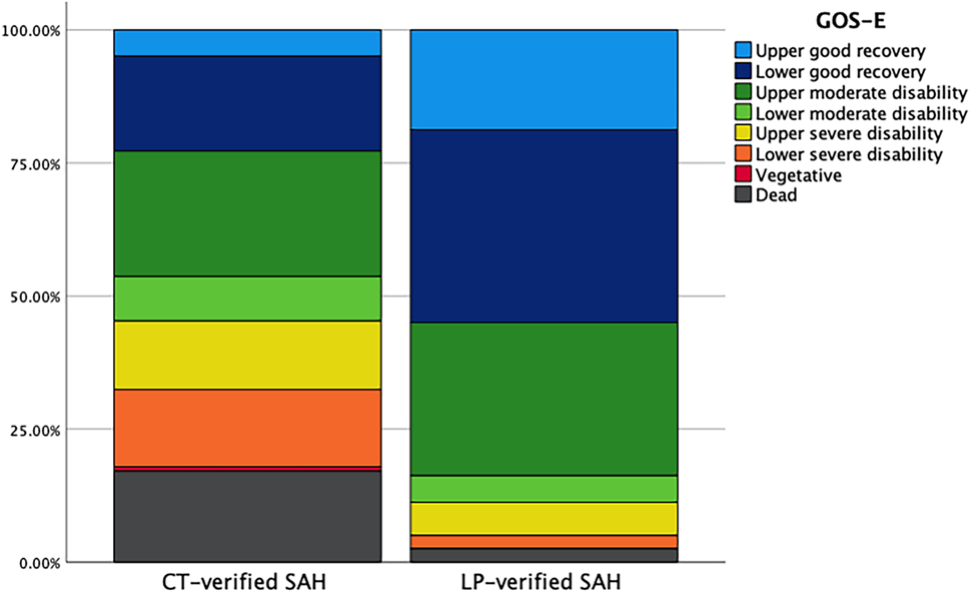
Clinical outcome for the CT- and LP-verified SAH cases 1-year post-ictus. The figure demonstrates clinical outcome (GOS-E) 12 months post-ictus for those with CT- and LP-verified SAH. Those with a CT-verified SAH had a higher rate of mortality (17% vs. 3%), vegetative state (1% vs. 0%), lower severe disability (15% vs. 3%), upper severe disability (13% vs. 6%), and lower moderate disability (8% vs. 5%), whereas they had a lower rate of upper moderate disability (24% vs. 29%), lower good recovery (18% vs. 36%), and upper good recovery (5% vs. 19%). CT = computed tomography. GOS-E = Glasgow Outcome Scale-Extended. LP = lumbar puncture. SAH = subarachnoid hemorrhage

At 1 year, the majority (89%) had recovered to a favorable functional outcome in the LP-verified SAH cohort, which was higher than in the CT-verified SAH cohort (55%, *p* < 0.001). Good recovery was reached in 55% of the LP-verified SAH cases (Fig. 2).

When the explanatory variables for clinical outcome were analyzed with univariate Spearman correlation in the cohort of LP-verified SAH cases (Table 4), those with a vascular pathology exhibited a lower GOS-E (*r* = -0.27, *p* = 0.02), as well as those with an EVD (*r* = -0.27, *p* = 0.02), but there were otherwise no association in this group between GOS-E and age, sex, length of stay in the NIC/neurointermediate care, time from ictus to diagnosis, WFNS grade, or rebleeding.

**Table 4:** Predictors of clinical outcome after LP-verified SAH – Spearman rank correlation test

Variables	GOS-E	
	*r*	*p*
Age (years)	-0.04	0.71
Sex (female)	-0.15	0.18
Length of stay (days)	-0.10	0.39
Time to diagnosis (days	-0.02	0.84
WFNS (grade)	-0.21	0.06
Vascular pathology (yes)	-0.27	***0.02***
Rebleeding (yes)	-0.11	0.32
External ventricular drainage (yes)	-0.27	***0.02***

Sex, 0 = male, 1 = female

Vascular pathology, 0 = no, 1 = yes, according to DSA

Rebleeding, 0 = no, 1 = yes

Length of stay = days spent in the NIC/neurointermediate care

Bold and italics indicate statistical significance

*DSA* digital subtraction angiography. *LP* lumbar puncture. *NIC* neurointensive care. *SAH* subarachnoid hemorrhage. *WFNS* World Federation of Neurosurgical Societies

## Discussion

In this study including 1280 spontaneous SAH patients, 80 patients exhibited a CT-negative LP-verified SAH and an underlying vascular pathology was found in almost one fifth of these patients. No risk factor was found for having an underlying vascular pathology in the LP-verified SAH group. Consistent with the previous SAH literature showing that patients with small initial bleedings exhibit a better functional recovery, almost 90% of the LP-verified SAH cases had a favorable recovery at 1 year. However, still, 45% of the cases did not reach good recovery, despite the small initial bleeding and a rather benign NIC course. This indicates that some of these LP-verified SAH patients warrant more attention and active neurological rehabilitation than what might have been anticipated.

### LP-verified SAH—frequency, patient characteristics, and clinical course

In this study, the patients with CT-negative, LP-verified SAH constituted 6% of the entire SAH cohort. Similar frequencies have been reported in other studies as well [20, 35, 44]. These patients were diagnosed at a later time point from onset of symptoms, as the time between ictus and CT-/LP-diagnosis was in median 3 rather than 0 days for the CT-verified cases. We do not have data explaining the cause of the delay. One explanation for the diagnostic delay could be that the initial bleeding did not elicit as severe symptoms, which led the patient to wait before seeking medical care [39]. Another explanation could be that some of these patients sought medical care in time, but due to doctor’s delay for various reasons the time to diagnosis was extended. If these patients would have sought care and done a CT at an earlier time point, the chances of it being positive would obviously have been higher since longer time from ictus allows the SAH to become isodense and CSF to dilute and flush away some of it [13, 22, 26, 40].

Furthermore, the LP-verified patients were younger, in a better neurological condition at admission (WFNS), exhibited a more benign NIC course with a lower rate of DIND and DC surgery, and recovered more favorably. This was highly expected and in agreement with the literature [31, 43] indicating that SAH patients with less visible blood on the initial CT exhibit a more favorable clinical course and recovery.

However, only 55% reached good functional outcome at 1 year. Having a vascular pathology and an EVD increased the risk of poor functional outcome, possibly partly related to rebleeding and treatment complications. In addition, although DIND did not occur in the LP-verified SAH group, some patients might still have suffered from silent brain infarctions [36]. All of these factors might have contributed to a worse outcome. It is also possible that LP-verified SAH in general was considered so benign that eventual problems, such as cognitive dysfunction and mood disturbances, were not sufficiently recognized and addressed. Consequently, these patients may warrant more follow-up and possibly neurological rehabilitation than what has been anticipated. Although information on the symptoms exhibited by the patient post SAH was not gathered in this study, it is known that, e.g., attention and memory deficits, chronic fatigue, and behavioral changes are common sequelae following aSAH [28]. It is likely that neurorehabilitation based on identifying the patient’s problems may be beneficial [21]. There are also some emerging adjunct therapies, such as repetitive transcranial magnetic stimulation, which may enhance neurological recovery after acute brain injuries [12].

### LP-verified SAH and underlying vascular pathologies—how should these patients be investigated?

In this study, the LP-verified SAH cases had a guilty aneurysm or AVM in 19% of the cases. The literature is relatively scarce on the rate of LP-verified SAH cases with an underlying vascular pathology. Previous studies have reported a wide range from 5 to 49% [3, 7, 8, 13, 19, 42, 48], which is likely explained by differences in the criteria for the LP-verified SAH diagnosis (visual inspection of xantochromia vs. biochemical analyses of CSF) and possibly differences in admission criteria and patient cohorts. No potential risk factors for finding a vascular pathology on DSA in the LP-verified SAH cohort were found in this study. This was also largely consistent with the limited number of previous studies [3, 8]. Only Bakker et al, found that female patients with LP-verified SAH were more prone to have an underlying aneurysm [3]. In our data, there was a trend towards longer time to diagnosis and the risk of having a positive DSA-finding, but several patients with LP-verified SAH diagnosed on day 0 and 1 were found to have a vascular pathology. Altogether it remains challenging to predict the rate of the LP-verified SAH patients that exhibit an aneurysm or AVM.

LP-verified SAH may be compared with perimesencephalic SAH, which is also a small bleeding, but extensive enough to be demonstrated on CT. Perimesencephalic SAH has a typical SAH distribution localized around the brainstem, which indicates a low-pressure bleeding, and is typically associated with negative vascular imaging studies and is rather explained by rupture of a small vein. LP-verified SAH may overlap with perimesencephalic SAH to some extent, e.g., for a patient with perimesencephalic SAH who waited a few days to seek medical care which allowed the CSF to flush and dilute the SAH making it no longer visible on CT. From a clinical point of view, the major difference with LP-verified and perimesencephalic SAH is that CT imaging does not reveal any hemorrhage epicenter in the former group, which makes it more challenging to guide the search for an underlying bleeding source on vascular imaging.

Altogether, our results showed that LP-verified SAH patients were less likely to have an underlying vascular pathology, but it still occurred in one fifth of the patients and no risk factor could be identified for this. All vascular pathologies were detected on both CTA and DSA among the LP-verified SAH patients. It has been reported that the sensitivity of a CTA to detect an aneurysm is around 95% and that those which are missed are usually small (below 5 mm) [24, 32]. DSA remains the gold standard [16], but it also carries some risks of complications such as local bleeding and stroke [1]. Thus, if the unenhanced CT was done within 24 hours from ictus and there were no particular risk factors for aneurysmal etiology (e.g., a combination of smoking, chronic arterial hypertension, heredity of SAH), and the CTA of high imaging quality was negative, we think this may be sufficient vascular work-up in case of LP-verified SAH. However, each case should be examined by a neurosurgeon and neurointerventionist together, taking into account the clinical risk factors of aneurysmal etiology and the quality of available vascular imaging, to decide whether a DSA is necessary [1, 17, 25].

### Methodological considerations

The main limitation of this study was the single-center retrospective design. We abstained from multiple logistic regressions due to the relatively limited number of LP-verified SAH cases (*n* = 80), and also because of the low number of DSA-positive cases (*n* = 15) in the cohort. However, the number of cases were still comparable to the relatively limited literature of LP-verified SAH patients [3, 7, 8, 42, 48]. Furthermore, we did not proceed with more in-depth analyses of LP-findings (e.g., the exact biochemical findings regarding erythrocytes, oxyhemoglobin, and bilirubin etc.), because these data were not consistently available since most of the LPs had been done at the local hospitals. The diagnosis of LP-verified SAH was based on the combination of clinical symptoms and LP-findings, however, it is possible that a few of these cases were false positives, e.g., due to increases in CSF bilirubin due to non-hemorrhagic conditions or technical laboratory issues [5]. The patients were followed up at 12 months post-ictus, which can be considered as a short period of time to evaluate functional outcome. A longer follow-up is to be preferred, but these data were not available. Furthermore, it is likely that the quality of the CT images differed among the patients, e.g., due to technical aspects and patient co-operation during imaging. This might have affected the chances of detecting SAH on CT to some extent. Lastly, it is possible that magnetic resonance imaging (MRI) including, e.g., FLAIR and SWI sequences that are sensitive to blood-break-down products might have been able to detect SAH in CT-negative cases and could then perhaps also have aided in localizing the epicenter of the bleeding [27, 49].

## Conclusions

Although LP-verified SAH cases more seldom exhibited an underlying vascular pathology, it was still found in almost one fifth of the patients, even for those who were CT-negative on day 0 and 1 post-ictus. No risk factor could be found for having an underlying vascular pathology. Almost 90% had recovered favorably at 1 year, but only 55% reached full recovery, despite the small bleeding and the generally benign NIC course. This indicates that these patients could favor from further neurological rehabilitation.

## Supplementary Information

Below is the link to the electronic supplementary material.

Supplementary file1 (DOCX 22 KB)

## Data Availability

Data are available upon reasonable request.

## References

[1] Agid R, Andersson T, Almqvist H, Willinsky RA, Lee S-K, terBrugge KG, Farb RI, Söderman M (2010). Negative CT angiography findings in patients with spontaneous subarachnoid hemorrhage: When is digital subtraction angiography still needed?. AJNR Am J Neuroradiol.

[2] Backes D, Rinkel GJE, Kemperman H, Linn FHH, Vergouwen MDI (2012). Time-dependent test characteristics of head computed tomography in patients suspected of nontraumatic subarachnoid hemorrhage. Stroke.

[3] Bakker NA, Groen RJM, Foumani M, Uyttenboogaart M, Eshghi OS, Metzemaekers JDM, Luijckx GJ, Van Dijk JMC (2014). Appreciation of CT-negative, lumbar puncture-positive subarachnoid haemorrhage: risk factors for presence of aneurysms and diagnostic yield of imaging. J Neurol Neurosurg Psychiatry.

[4] Bederson JB, Connolly ES, Batjer HH (2009). Guidelines for the management of aneurysmal subarachnoid hemorrhage: a statement for healthcare professionals from a special writing group of the Stroke Council American Heart Association. Stroke.

[5] Beetham R (2009). CSF spectrophotometry for bilirubin–why and how?. Scand J Clin Lab Invest.

[6] Boesiger BM, Shiber JR (2005) Subarachnoid hemorrhage diagnosis by computed tomography and lumbar puncture: are fifth generation CT scanners better at identifying subarachnoid hemorrhage? J Emerg Med 29(1):23–27. https://www.sciencedirect.com/science/article/pii/S0736467905000673casa_token=Rt1Tn74nGY0AAAAA:8N3f6WLUIiAh6cHnTf5ykwD7oYvd9YPJ82tzQ5AujH8uHiPwKPWDU2xjrPLNEq_vqRV6NbqKw 10.1016/j.jemermed.2005.02.002 15961003

[7] Carstairs SD, Tanen DA, Duncan TD, Nordling OB, Wanebo JE, Paluska TR, Theodore N, Riffenburgh RH (2006). Computed tomographic angiography for the evaluation of aneurysmal subarachnoid hemorrhage. Acad Emerg Med Off J Soc Acad Emerg Med.

[8] Chalouhi N, Witte S, Penn DL, Soni P, Starke RM, Jabbour P, Gonzalez LF, Dumont AS, Rosenwasser R, Tjoumakaris S (2013). Diagnostic yield of cerebral angiography in patients with computed tomography-negative, lumbar puncture-positive subarachnoid hemorrhage. Neurosurgery.

[9] Connolly ES, Rabinstein AA, Carhuapoma JR (2012). Guidelines for the management of aneurysmal subarachnoid hemorrhage: a guideline for healthcare professionals from the American Heart Association/american Stroke Association. Stroke.

[10] Cortnum S, Sørensen P, Jørgensen J (2010). Determining the sensitivity of computed tomography scanning in early detection of subarachnoid hemorrhage. Neurosurgery.

[11] de Rooij NK, Linn FHH, van der Plas JA, Algra A, Rinkel GJE (2007). Incidence of subarachnoid haemorrhage: a systematic review with emphasis on region, age, gender and time trends. J Neurol Neurosurg Psychiatry.

[12] Dionísio A, Duarte IC, Patrício M, Castelo-Branco M (2018). The Use of Repetitive Transcranial Magnetic Stimulation for Stroke Rehabilitation: A Systematic Review. J Stroke Cerebrovasc Dis Off J Natl Stroke Assoc.

[13] Ditta M, Galea J, Holland J, Patel HC (2013). Lumbar puncture and the diagnosis of CT negative subarachnoid haemorrhage: time for a new approach?. Br J Neurosurg.

[14] Enblad P, Persson L (1997). Impact on clinical outcome of secondary brain insults during the neurointensive care of patients with subarachnoid haemorrhage: a pilot study. J Neurol Neurosurg Psychiatry.

[15] Engquist H, Rostami E, Ronne-Engström E, Nilsson P, Lewén A, Enblad P (2018). Effect of HHH-Therapy on Regional CBF after Severe Subarachnoid Hemorrhage Studied by Bedside Xenon-Enhanced CT. Neurocrit Care.

[16] Gonzalez AM, Narata AP, Yilmaz H, Bijlenga P, Radovanovic I, Schaller K, Lovblad K-O, Pereira VM (2014). Blood blister-like aneurysms: single center experience and systematic literature review. Eur J Radiol.

[17] Heit JJ, Pastena GT, Nogueira RG, Yoo AJ, Leslie-Mazwi TM, Hirsch JA, Rabinov JD (2016). Cerebral Angiography for Evaluation of Patients with CT Angiogram-Negative Subarachnoid Hemorrhage: An 11-Year Experience. AJNR Am J Neuroradiol.

[18] Ingall T, Asplund K, Mähönen M, Bonita R (2000). A multinational comparison of subarachnoid hemorrhage epidemiology in the WHO MONICA stroke study. Stroke.

[19] Kameda-Smith M, Aref M, Jung Y, Ghayur H, Farrokhyar F (2021). Determining the Diagnostic Utility of Lumbar Punctures in Computed Tomography Negative Suspected Subarachnoid Hemorrhage: A Systematic Review and Meta-analysis. World Neurosurg.

[20] Langham J, Reeves BC, Lindsay KW (2009). Variation in outcome after subarachnoid hemorrhage: a study of neurosurgical units in UK and Ireland. Stroke.

[21] Lindner A, Brunelli L, Rass V (2023). Long-Term Clinical Trajectory of Patients with Subarachnoid Hemorrhage: Linking Acute Care and Neurorehabilitation. Neurocrit Care.

[22] Mark DG, Sonne DC, Jun P, Schwartz DT, Kene MV, Vinson DR, Ballard DW (2016). False-negative Interpretations of Cranial Computed Tomography in Aneurysmal Subarachnoid Hemorrhage. Acad Emerg Med Off J Soc Acad Emerg Med.

[23] McCormack RF, Hutson A (2010). Can computed tomography angiography of the brain replace lumbar puncture in the evaluation of acute-onset headache after a negative noncontrast cranial computed tomography scan?. Acad Emerg Med Off J Soc Acad Emerg Med.

[24] Menke J, Larsen J, Kallenberg K (2011). Diagnosing cerebral aneurysms by computed tomographic angiography: meta-analysis. Ann Neurol.

[25] Mohan M, Islim A, Dulhanty L, Parry-Jones A, Patel H (2019). CT angiogram negative perimesencephalic subarachnoid hemorrhage: is a subsequent DSA necessary? A systematic review. J Neurointerventional Surg.

[26] Mohan M, Islim AI, Rasul FT (2019). Subarachnoid haemorrhage with negative initial neurovascular imaging: a systematic review and meta-analysis. Acta Neurochir (Wien).

[27] Nelson SE, Sair HI, Stevens RD (2018). Magnetic Resonance Imaging in Aneurysmal Subarachnoid Hemorrhage: Current Evidence and Future Directions. Neurocrit Care.

[28] Nussbaum ES, Mikoff N, Paranjape GS (2021). Cognitive deficits among patients surviving aneurysmal subarachnoid hemorrhage. A contemporary systematic review. Br J Neurosurg.

[29] Nyholm L, Howells T, Enblad P, Lewén A (2013). Introduction of the Uppsala Traumatic Brain Injury register for regular surveillance of patient characteristics and neurointensive care management including secondary insult quantification and clinical outcome. Ups J Med Sci.

[30] Peerless SJ (1979). Pre- and postoperative management of cerebral aneurysms. Clin Neurosurg.

[31] Pegoli M, Mandrekar J, Rabinstein AA, Lanzino G (2015). Predictors of excellent functional outcome in aneurysmal subarachnoid hemorrhage. J Neurosurg.

[32] Philipp LR, McCracken DJ, McCracken CE (2017). Comparison Between CTA and Digital Subtraction Angiography in the Diagnosis of Ruptured Aneurysms. Neurosurgery.

[33] Ryttlefors M, Howells T, Nilsson P, Ronne-Engström E, Enblad P (2007). Secondary insults in subarachnoid hemorrhage: occurrence and impact on outcome and clinical deterioration. Neurosurgery.

[34] Sames TA, Storrow AB, Finkelstein JA, Magoon MR (1996). Sensitivity of new-generation computed tomography in subarachnoid hemorrhage. Acad Emerg Med Off J Soc Acad Emerg Med.

[35] Sayer D, Bloom B, Fernando K, Jones S, Benton S, Dev S, Deverapalli S, Harris T (2015). An Observational Study of 2,248 Patients Presenting With Headache, Suggestive of Subarachnoid Hemorrhage, Who Received Lumbar Punctures Following Normal Computed Tomography of the Head. Acad Emerg Med Off J Soc Acad Emerg Med.

[36] Schmidt JM, Wartenberg KE, Fernandez A, Claassen J, Rincon F, Ostapkovich ND, Badjatia N, Parra A, Connolly ES, Mayer SA (2008). Frequency and clinical impact of asymptomatic cerebral infarction due to vasospasm after subarachnoid hemorrhage. J Neurosurg.

[37] Suarez JI (2015). Diagnosis and Management of Subarachnoid Hemorrhage. Contin Minneap Minn.

[38] Svedung Wettervik T, Howells T, Lewén A, Ronne-Engström E, Enblad P (2021). Temporal Dynamics of ICP, CPP, PRx, and CPPopt in High-Grade Aneurysmal Subarachnoid Hemorrhage and the Relation to Clinical Outcome. Neurocrit Care.

[39] Tetsuka S, Matsumoto E (2016). Diagnosis of a subarachnoid hemorrhage with only mild symptoms using computed tomography in Japan. BMC Neurol.

[40] Tong D-M, Zhou Y-T (2010). Predictors of the subarachnoid hemorrhage of a negative CT scan. Stroke.

[41] Tulla M, Tillgren T, Mattila K (2019). Is there a role for lumbar puncture in early detection of subarachnoid hemorrhage after negative head CT?. Intern Emerg Med.

[42] van den Berg R, Jeung L, Post R, Coert BA, Hoogmoed J, Coutinho JM, Majoie CB, Verbaan D, Emmer BJ, Vandertop WP (2021) The added value of cerebrospinal fluid analysis in patients with subarachnoid hemorrhage after negative noncontrast CT. J Neurosurg 1–5 10.3171/2021.4.JNS21656 34560662

[43] van Donkelaar CE, Bakker NA, Birks J, Veeger NJGM, Metzemaekers JDM, Molyneux AJ, Groen RJM, van Dijk JMC (2019). Prediction of Outcome After Aneurysmal Subarachnoid Hemorrhage. Stroke.

[44] van Gijn J, Kerr RS, Rinkel GJE (2007). Subarachnoid haemorrhage. Lancet Lond Engl.

[45] van Gijn J, Rinkel GJ (2001). Subarachnoid haemorrhage: diagnosis, causes and management. Brain J Neurol.

[46] van Heuven AW, Dorhout Mees SM, Algra A, Rinkel GJE (2008). Validation of a prognostic subarachnoid hemorrhage grading scale derived directly from the Glasgow Coma Scale. Stroke.

[47] Vårdförlopp stroke och TIA. https://skr.se/kunskapsstyrningvard/kunskapsstod/publiceradekunskapsstod/nervsystemetssjukdomar/vardforloppstrokeochtia.62398.html. Accessed 27 Feb 2023

[48] Wallace AN, Dines JN, Zipfel GJ, Derdeyn CP (2013). Yield of catheter angiography after computed tomography negative, lumbar puncture positive subarachnoid hemorrhage [corrected]. Stroke.

[49] Woodcock RJ, Short J, Do HM, Jensen ME, Kallmes DF (2001). Imaging of acute subarachnoid hemorrhage with a fluid-attenuated inversion recovery sequence in an animal model: comparison with non-contrast-enhanced CT. AJNR Am J Neuroradiol.

