# Supplementary material for: Lumbar puncture-verified subarachnoid hemorrhage: bleeding sources, need of radiological examination, and functional recovery
Source: Acta Neurochir (Wien). 2023 May 25;165(7):1847–54. doi: 10.1007/s00701-023-05640-4 (PMC10319674; doi:10.1007/s00701-023-05640-4)
Supplement: Supplementary file 1 — Supplementary file1 (DOCX 22 KB) [file 701_2023_5640_MOESM1_ESM.docx]

**Fig.1 Flowchart of patient inclusion**

**1325** patients with spontaneous SAH

**45** patients excluded

33 Treated at another NICU >3 days during the first 10 days

11 Missing data

1 <16 years

**1280** patients included

There were 1325 patients with spontaneous SAH, treated between January 7^th^ 2008 and April 9^th^ 2018, who were eligible for inclusion in this study. For the 1325 patients, 45 were excluded; 33 patients were treated at another NIC unit for more than 3 days during the first 10 days, 11 patients had missing clinical and radiological data, and 1 patient was younger than 16 years of age. The final study cohort was hence 1280 SAH patients.

CT = Computed tomography. LP = Lumbar puncture. NIC = Neurointensive care. SAH = Subarachnoid hemorrhage.
